# Supplementary material for: MetaRibo-Seq measures translation in microbiomes
Source: Nat Commun. 2020 Jun 29;11:3268. doi: 10.1038/s41467-020-17081-z (PMC7324362; doi:10.1038/s41467-020-17081-z)
Supplement: Supplementary file 10 — Supplementary Data 7 [file 41467_2020_17081_MOESM10_ESM.zip › File2/Confidence_High_Taxonomy/221006_out.krona.html]

Javascript must be enabled to view this page.

members
magnitude
magnitudeUnassigned
count
unassigned
taxon
rank

221006\_out

5

2
superkingdom
5

1239
phylum
5

186801
class
5

order
186802
5

5
family
541000

39492

SRS011134\_contig\_number\_22966SRS014459\_contig\_number\_41399SRS019161\_contig\_number\_contig-100\_1052.238318SRS022609\_contig\_number\_contig-100\_7109.171272SRS063190\_contig\_number\_13296
species
5
